# Degradation of Steel Rebar Tensile Properties Affected by Longitudinal Non-Uniform Corrosion

**DOI:** 10.3390/ma16072917

**Published:** 2023-04-06

**Authors:** Jinhong Liu, Xiaoyong Luo, Qi Chen

**Affiliations:** School of Civil Engineering, Central South University, Changsha 410075, China; jinhongl@csu.edu.cn (J.L.);

**Keywords:** steel rebar, longitudinal non-uniform corrosion, tensile properties, 3D laser scanning, cross-sectional area, mechanisms

## Abstract

Rebar corrosion is the primary cause of the durability degradation of reinforced concrete (RC) structures, where non-uniform corrosion is the typical pattern in engineering. This study experimentally and numerically investigated the tensile degradation properties of non-uniform corroded rebars. Corrosion morphology was accurately determined by three-dimensional (3D) laser scanning techniques, studying the characteristics of longitudinal non-uniform corrosion. The results showed that the non-uniformity of corrosion increased with an increase in corrosion levels. From tensile tests, the differences in nominal stress–strain curves among rebars with similar average corrosion levels indicated that corrosion non-uniformity has appreciable effects on the tensile behavior of rebars. The residual load-bearing capacity of corroded rebars was dominated by the reduced critical cross-section, while residual ductility was associated with the cross-section loss throughout the entire length of rebars. The degradation relations of nominal yield and ultimate strength, ultimate strain, and elongation after fracture were better correlated to the maximum cross-section loss than to the average volume loss. Additionally, numerical calculation based on the cross-sectional areas of corroded rebars was conducted to evaluate the tensile behavior of non-uniform corroded rebars. Equivalent distribution models simulating the longitudinal non-uniform corrosion were proposed, on the basis of probability characteristics of cross-sectional areas, for practical application of the numerical method.

## 1. Introduction

Reinforced concrete (RC) is widely used in the construction of civil infrastructures worldwide. However, corrosion of embedded steel rebars is a major problem faced by a great number of RC structures that limits their service life. Several studies have indicated that corrosion decreases the tensile properties of steel rebars, causes concrete cracking and spalling [1,2], and affects the bond behavior between the steel rebar and concrete [3,4]. Performance degradation not only concerns the safety of structures [5,6], but may also lead to high economic costs and negative environmental impacts [7]. Therefore, it is essential to properly estimate the degraded properties of corroded rebars in the maintenance of existing structures and the sustainable design of new structures.

Several experimental studies in the past decade have investigated the effect of corrosion on the tensile behavior of corroded rebars, from which some commonly recognized conclusions can be made that corrosion causes a remarkable reduction in the load-bearing capacity and ductility of corroded rebars [8,9,10,11]. Moreover, several researchers have proposed degradation formulae to estimate the tensile properties of corroded rebars, as summarized in Table 1. Tested rebars were obtained from naturally corroded ex-service RC structures, artificial corrosion programs in cyclic wet and dry conditions, and electrical accelerated corrosion programs. Load-bearing capacity, mainly represented by the yield and ultimate load [10,12] and nominal strength [9,13,14,15,16,17,18,19,20] decrease linearly with an increase in the corrosion degree. For ductility, however, the decay laws show some difference in the equation form even though the same performance indices were utilized: linear function [13,16,17,18,19,20] and exponential function [9,10,12,14] were proposed. The degradation factors for both the load-bearing capacity and the ductility indices of corroded rebars also differ markedly from one study to another, such as the fact that the degradation factors of the nominal yield and ultimate strength in linear equations are 0.0110 to 0.0210 and 0.0107 to 0.0231, respectively.

Apart from the influence of the rebar type and size [10], various corrosion inducements and processes contributed to the significant diversity in performance degradation formulae of the corroded rebars [8,18,20]. Aggressive ions penetration, passive film destruction, and corrosion propagation are a series of complex physical and chemical processes affected by the external environment and material composition [21,22]. As a result, corrosion patterns vary with diverse corrosion processes and conditions [23,24], and apparent differences in degradation models based on test results can be observed even in studies where rebar specimens were obtained by similar corrosion tests with few different environmental conditions. However, most existing degradation models were empirical models that were purely established by regression analysis based on test results and were simply applicable to specific rebar types and exposure conditions. Although some theoretical analysis has been performed [25], it has simply assumed that the corrosion only leads to a reduction in cross-sections, which needs to be studied in depth. In order to break the limitation for largescale practical applications of traditional models and further utilize existing precious samples to build stochastic corrosion databases, the corrosion-related degradation mechanisms of tensile properties remain to be further investigated and taken into account in the estimation.

On the other hand, numerous researchers have adopted mass or volume loss as the characterization of corrosion to estimate the degraded tensile properties of corroded rebars, which represent the average corrosion level of whole rebars, but have not considered the effects of corrosion patterns and localized severe corrosion. The corrosion of rebars can be classified as chloride-induced corrosion [26,27] and carbonation-induced corrosion [22], of which the corrosion patterns are generally considered to be pitting and uniform, respectively. Nevertheless, owing to the non-uniform composition of concrete and steel, random initial defects, reinforcement placing in structures, loading cracks, and varying environmental conditions, corrosion in RC structures is a stochastic field problem with probabilistic spatiotemporal distributions. Absolutely uniform corrosion is an ideal state that hardly exists in engineering. It is often observed that corrosion on the rebar surface is longitudinally and circumferentially non-uniform [28,29]. Furthermore, corrosion patterns influence the tensile properties of corroded rebars [30]. Ignoring corrosion pattern effects may reduce the accuracy of the degraded performance models of corroded rebars. Due to the difficulty in accurately measuring residual corrosion morphology with traditional methods, there have been few studies [11,31] on longitudinal non-uniform corrosion and its effects on the tensile properties of rebars.

Researchers focusing on the stochasticity of corrosion have made efforts to establish stochastic models of steel bars [32] and structural steel [33]. Corrosion randomness was also considered in the reliability analysis [34] and reliability-based design optimization [35]. Nevertheless, the study on random field problems requires a large number of samples and to consider many variables. A better understanding of the non-uniform corrosion effects and degradation mechanisms of tensile properties will be helpful for decreasing variables and increasing the study efficiency and accuracy.

Thus, the present study investigates the degraded tensile properties of rebars under the influence of longitudinal non-uniform corrosion with 3D laser scanning techniques. First, 45 non-uniform corroded rebars were obtained by a modified artificial corrosion experiment for several pre-cracked and intact RC slabs. Then, using 3D laser scanning techniques, residual cross-sectional areas along the length of rebars were determined, and non-uniform corrosion characteristics were explored. Subsequently, the degraded tensile behavior of non-uniform corroded rebars was studied by tensile tests. With the aid of numerical analysis, the degradation mechanisms were also investigated. Finally, considering the effect of longitudinal non-uniform corrosion, degradation models and a numerical method based on equivalent cross-section distribution models were proposed to estimate the load-bearing capacity and ductility of corroded rebars.

## 2. Experimental Program

### 2.1. Specimen Design and Preparation

**Figure 1:**
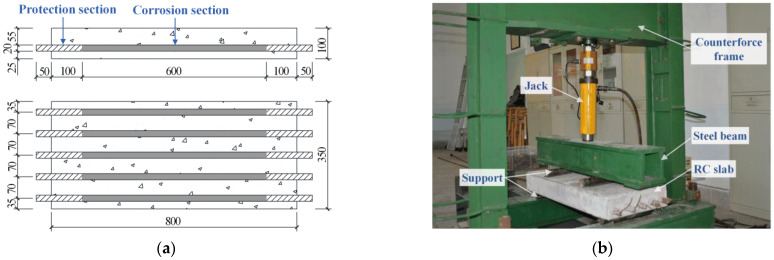
RC specimen design and preparation: (**a**) slab dimensions and steel rebar layout (unit: mm); (**b**) loading application.

In this study, nine 800 mm × 350 mm × 100 mm reinforced concrete slabs were prepared for accelerated corrosion. The studied rebar, HRB400 (hot-rolled ribbed steel rebar with a yield strength greater than 400 MPa and an ultimate strength greater than 540 MPa), is typically used in China. The steel rebar specimens had a nominal diameter of 20 mm. Five rebars, 900 mm in length, were partially embedded in one slab with a cover thickness of 25 mm, and the two ends of each rebar extended for 50 mm beyond the slab to connect electric wires, as shown in Figure 1a. In order to avoid rebar fractures within clamps during tensile testing, two gripped ends with a length of 150 mm were coated with epoxy resin as protection sections. The middle section of approximately 600 mm was subjected to corrosion. A total of 53 specimens, including 45 corroded and 8 uncorroded rebars from eight pieces of parent materials, were prepared. The average measured mechanical properties of eight uncorroded rebars are shown in Table 2. After RC slab casting and 28 days of indoor nature curing, four slabs were pre-cracked at the bottom by loading two concentrated forces at quarter points to simulate concrete defects and bending cracks in engineering (see Figure 1b). The loading was carried out step by step according to the GB/T 50152-2012 Standard [36] until the maximum crack width reached 2 mm. Crack development was monitored with a crack observer between each loading stage. It should be stated that pre-cracking in this study is considered to be a cause of various corrosion morphologies and that the quantitative effect of cracks is beyond the scope of this study.

### 2.2. Accelerated Corrosion Procedure

**Figure 2:**
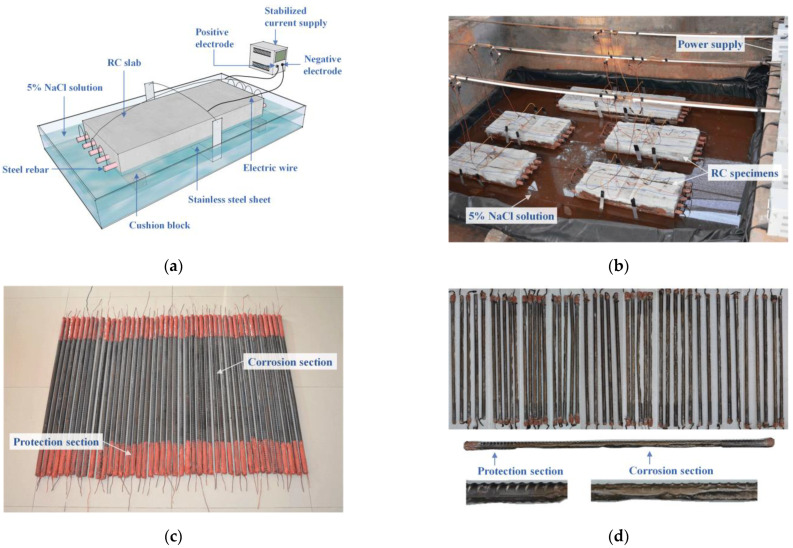
Accelerated corrosion set-up and specimens: (**a**) schematic illustration of accelerated corrosion set-up; (**b**) test instrumentation of half-immersed slabs; (**c**) rebar specimens for accelerated corrosion; (**d**) corroded rebars after rust cleaning.

Corroded rebars were acquired by an artificial accelerated corrosion process, in which rebars embedded in concrete slabs were subjected to impressed direct current. According to Wang’s test [24], the half-immersion method led to severe non-uniform corrosion. Thus, RC slabs were horizontally placed in a pool filled with 5% NaCl solution so that the slabs were partially immersed and the steel rebars were kept above the liquid level, as shown in Figure 2. Because the side near the cathode will be corroded more seriously [37], stainless steel sheets used as cathodes were distributed around the concrete cover side and connected to the negative end of the DC supply, to simulate one-side corrosion in residual engineering conditions. The applied current density was 300 μA/cm^2^ constantly during the accelerated corrosion. The predefined mass loss was from 5% to 25%, and the corresponding required time duration was evaluated using Faraday’s law. After the expected corrosion times, the corroded rebars were extracted from the concrete slabs. Corrosion products on rebars were removed by a steel brush and chemical pickling. The final corroded rebar specimens are shown in Figure 2d.

### 2.3. 3D Laser Scanning

The surface morphology of 53 rebars was measured using a 3D laser scanner (*Handyscan 700*, by CREAFORM, Canada) with a measuring accuracy of 0.03 mm. Compared with traditional methods, 3D laser scanning has the characteristics of non-contact, high measuring accuracy, and high efficiency, which are applicable to obtaining the complex corrosion pattern of steel rebars. After acquiring the 3D coordinates of each point on the rebar surface by scanning, *Geomagic Studio* software was used to optimize the data and form point cloud files with high signal-to-noise ratio data. Curved surfaces of rebars were then created by *VXelements* software (version 6.1, by CREAFORM, Canada), and 3D solid models were finally built in *Pro/ENGINEER* software (version Wildfire 5.0, by PTC, USA).

In order to explore the corrosion distribution and estimate the corrosion-induced cross-section loss and volume loss of rebars, discrete cross-sectional areas were obtained at 1 mm intervals along the longitudinal axis of each rebar 3D model in *Pro/ENGINEER* software. The residual volume *V* can be calculated using Equation (1) based on the second order Newton–Cotes formula:

(1) $$V=\sum_{i=1}^{l/h}\frac{h}{6}\left(A_{2i-1}+4A_{2i}+A_{2i+1}\right)$$

where *l* is the calculated length of the rebar, which is divided into microsegments with a length of *h*, i.e., in the present paper *h* = 2 mm, and totally *l*/*h* segments were divided. *A*_2*i*−1_, *A*_2*i*+1_, and *A*_2*i*_ are the cross-sectional area of two ends and the middle of the *i*th microsegment, respectively. To study the corrosion effect on the tensile behavior of rebars exactly, the calculated length *l* equals the actual parallel length in tensile tests.

Then, the average cross-section loss *η*_avg_, maximum cross-section loss *η*_max_, and the average volume loss *η*_V_ of each rebar can be calculated as:

(2) $$\eta_{\mathrm{avg}}=\frac{A_{0,\mathrm{avg}}-A_{c,\mathrm{avg}}}{A_{0,\mathrm{avg}}}\times 100\%$$

(3) $$\eta_{\max}=\frac{A_{0,\min}-A_{c,\min}}{A_{0,\min}}\times 100\%$$

(4) $$\eta_{V}=\frac{V_{0}-V_{c}}{V_{0}}\times 100\%$$

where *V*_0_ is the volume of the uncorroded rebar with the same length as the studied specimen, calculated by the following equation based on the volume of eight uncorroded specimens:

(5) $$V_{0}=(\frac{1}{8}\sum_{i=1}^{8}\frac{V_{0i}}{l_{0i}})\times l_{c}$$

Finally, rebar specimens were numbered in the order of volume loss from small to large (see Table 3), where U represents uncorroded rebars, L represents corroded rebars from pre-cracked slabs, and J represents corroded rebars from intact slabs. It can be seen from Table 3 that the average cross-section loss almost equaled the average volume loss, indicating that both of them can reflect the average corrosion level of steel rebars.

### 2.4. Tensile Testing

**Figure 3:**
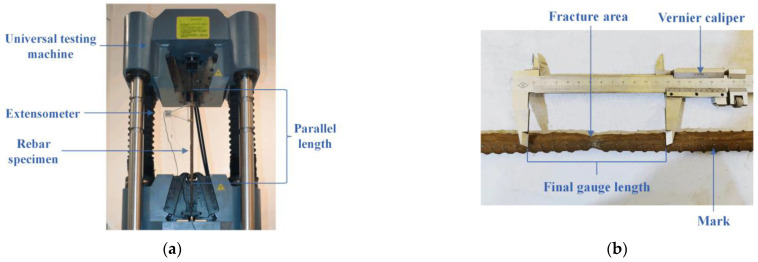
Tensile test set-up and testing: (**a**) loading arrangement; (**b**) measuring elongation.

Rebar specimens were subjected to uniaxial tensile testing according to the standard GB/T228.1-2010 [38], which referred to ISO 6892-1: 2009, using an electro-hydraulic servo universal testing machine with a strength capacity of 1000 kN, as shown in Figure 3. Sections 60 mm in length at each end of the specimens were cut off before testing to avoid a rebar fracture within clamps resulting from local corrosion at the concrete slab and air boundary. Marks on the rebar surface were made at a distance of 10 mm to identify the original gauge length, which is five times the nominal uncorroded diameter, i.e., the distance between 11 marks. The monotonic tensile process was controlled by the strain rate, with a normal speed of 0.00025/s before yielding and 0.0067/s at the plastic stage. During tests, extensions of rebars at the elastic zone were measured by an extensometer with a gauge length of 100 mm. The applied load and displacement data were recorded using an automatic data acquisition system and were used to plot the load-displacement curve. Post failure, the final gauge length was measured by a vernier caliper between 11 markers near the fracture area to calculate the elongation after fracture, which is the ratio of the elongation to the original gauge length. The yield load, ultimate load, and elongation of each rebar were determined based on the corresponding test.

## 3. Corrosion Characteristics

### 3.1. Corrosion Pattern

**Figure 4:**
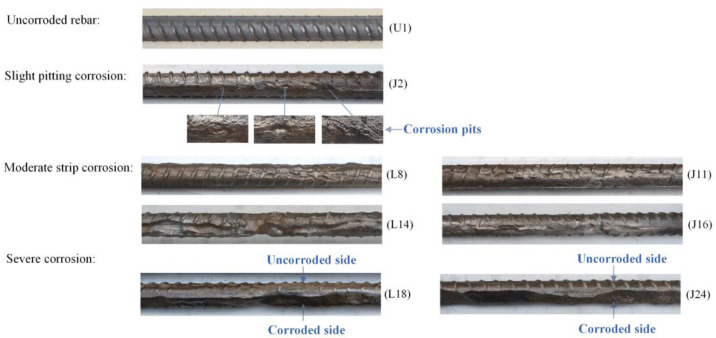
Corrosion patterns of rebars with different corrosion levels.

Figure 4 shows representative corrosion patterns of uncorroded and corroded rebars with different corrosion levels from the accelerated corrosion experiment. It can be seen that the specimens were non-uniformly corroded in the circumferential direction, i.e., radially one side was observably more corroded than other sides, consistent with corrosion patterns in engineering [39]. Furthermore, corrosion was also non-uniformly distributed along the length of rebars. Slight pit corrosion was a common pattern when the corrosion level was still low. As the corrosion progressed, pits deepened, widened, and connected gradually, resulting in irregular strip corrosion. When the average volume loss was greater than 20%, the corroded region almost covered the entire length of the rebar, and the corrosion depth was so great that cross-sections changed from circular to semicircular.

### 3.2. Longitudinal Non-Uniform Corrosion

**Figure 5:**
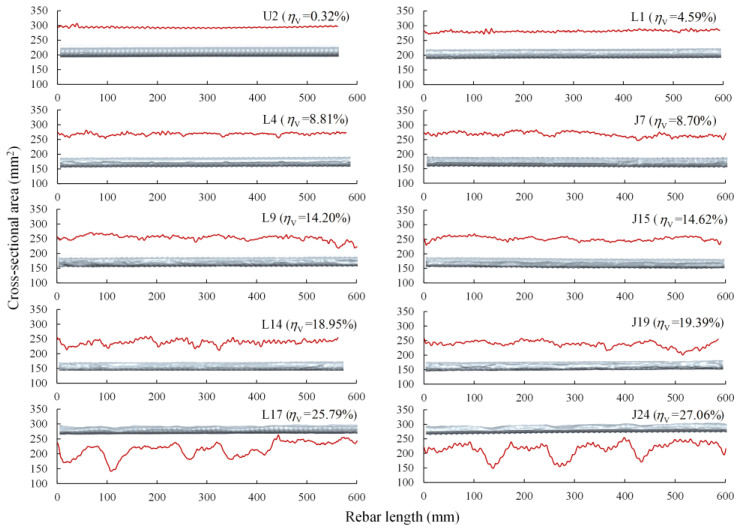
Residual cross-sectional areas along the length of rebar and rebar 3D solid models.

The residual cross-sectional area reflecting the multi-dimensional corrosion loss on the whole circumference is more comprehensive than the corrosion depth and more straightforward than other corrosion indices [28]. To better understand the longitudinal corrosion distribution of steel rebars, Figure 5 shows the residual cross-sectional areas along the length of steel rebars with different corrosion levels within the parallel length. It can be observed that, affected by transverse ribs, cross-sectional areas of the uncorroded rebar fluctuated slightly and regularly along the rebar length, such as specimen U2 shown in Figure 5. Except for the influence of transverse ribs, valleys in the figure represent the cross-sections of corroded regions, and their depth and length reflect the localized corrosion level and corrosion range, respectively. For corroded rebars from both pre-cracked and intact slabs, random forms of cross-sectional areas were observed, indicating that corrosion was distributed unevenly along the length of the rebars, and the localized corrosion degree and range were stochastic. As the average volume loss increased, the fluctuation in the cross-sections became more drastic. For steel rebars with an average volume loss greater than 20%, visible valleys were seen at the position of severe local corrosion, such as specimens L17 and J24 in Figure 5.

**Figure 6:**
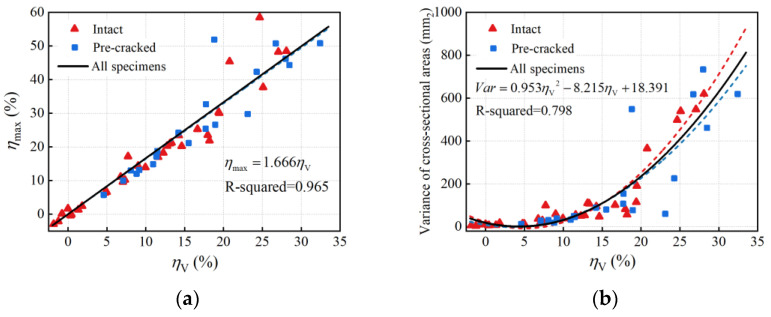
Non-uniform corrosion parameters: (**a**) maximum cross-section loss; (**b**) variance in cross-sectional areas.

The maximum cross-section loss and variance of cross-sectional areas of 53 rebar specimens were obtained to characterize the non-uniformity of longitudinal corrosion. The maximum cross-section loss calculated by Equation (3) reflects the most severe localized corrosion degree over the entire rebar. Figure 6a shows the relationship between the maximum cross-section loss and the average volume loss. As the average volume loss increased, the maximum cross-section loss increased linearly, indicating that the localized corrosion became more severe with the increasing average corrosion degree. This means that the deviation between localized and average corrosion levels increases, and the longitudinal corrosion non-uniformity intensifies.

The variance in cross-sectional areas (*Var*) represents the dispersion of cross-sections over the entire rebar.

Figure 6b shows the variance in cross-sectional areas of 53 rebars. It can be observed that the variance in cross-sectional areas tended to increase nonlinearly at a faster rate as the average volume loss increased, which is a clear indication of increasing longitudinal non-uniformity of corrosion with an increase in average corrosion degree. A quadratic polynomial function was proposed by regression analysis using the least square method to describe the relationship between the variance in cross-sectional areas and the average volume loss. When the average volume loss was greater than 20%, the data points in Figure 6 were distributed discretely; this was due to the increase in corrosion non-uniformity.

**Figure 7:**
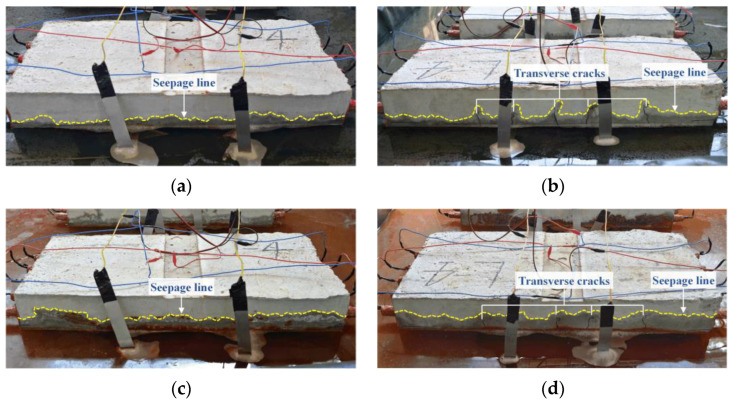
Corrosion procedure: (**a**) intact slab at initial stage; (**b**) pre-cracked slab at initial stage; (**c**) intact slab at middle stage; (**d**) pre-cracked slab at middle stage.

In the present paper, longitudinal non-uniform corrosion was observed on corroded rebars from both pre-cracked and intact slabs. It should be mentioned that there is little difference in corrosion non-uniformity between the two kinds of RC slabs in the experiment. The reasons may be as follows: On the one hand, the corrosion morphology of reinforcement is affected by numerous factors, such as the heterogeneity of the concrete microstructure (random distribution of coarse aggregate, pores, and initial defects) [40,41], the stochasticity of steel material [37], reinforcement placements [42], loading cracks [17], and rust-expansion-induced cracks. Bending cracks are not the only factor leading to uneven corrosion. On the other hand, the impressed field greatly accelerates the chloride ion migration rate over the entire length of the concrete slabs [20], reducing the difference originally in the engineering corrosion between cracking and non-cracking zones of RC slabs. As shown in Figure 7, the NaCl solution penetrated faster into the cracking zones of the pre-cracked slab initially, while pre-loading cracks had little influence on the erosion depth in the subsequent accelerated corrosion process. Furthermore, the seepage line of the intact slab was an irregular fluctuating curve, reflecting the influence of other factors on the erosion, except for the loading cracks. Hence, subsequent studies on tensile properties will take the slab type out of consideration.

### 3.3. Probability Distribution of Cross-Sectional Areas

**Figure 8:**
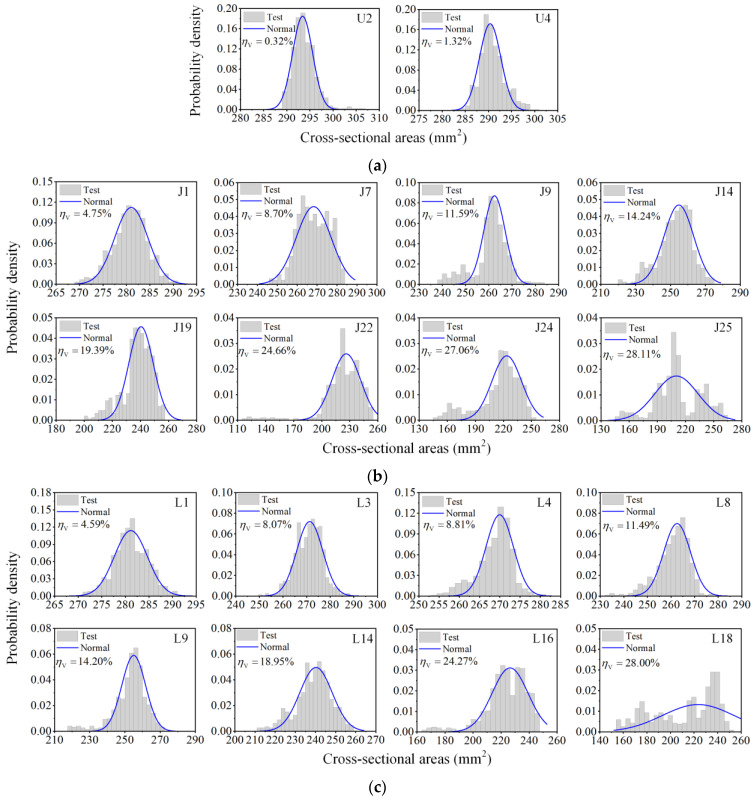
Probability distribution of cross-sectional areas: (**a**) uncorroded rebars; (**b**) rebars from intact slab; (**c**) rebars from pre-cracked slab.

According to the above analysis, the residual surface of the corroded steel rebars is irregular, and corrosion occurs unevenly along the length of the rebar. To explore the statistics of longitudinal non-uniformity of corrosion, the distribution of cross-sectional areas of 53 rebar specimens was investigated. Probability distribution histograms of cross-sectional areas of specimens with various volume losses are demonstrated in Figure 8. For uncorroded rebars and corroded rebars with low average corrosion levels, cross-sectional areas approximately followed a symmetrical unimodal distribution (i.e., specimens U2, U4, J1, L1). With an increase in average volume loss, the distribution of cross-sectional areas gradually changed into a single-peak left-skewed pattern (i.e., specimens J9, J14, J19, L8, L9, L14), which was attributed to severe corrosion within some limited regions. In general, the higher the average volume loss was, the longer the left tail of the distribution would be. As shown in Figure 8, the left tails of specimens J22 and L16 (with *η*_V_ of 24.66% and 24.27%) were significantly longer than those of J14 and L9 (with *η*_V_ of 14.24% and 14.20%). When the average volume loss was greater than 20%, the distribution of residual cross-sectional areas could appear as multimodal patterns (i.e., specimens J24, J25, L18) due to the significant non-uniformity of longitudinal corrosion resulting from high corrosion levels. The cross-sectional area distribution of rebars from pre-cracked and intact slabs followed the same trend with the growing average corrosion level.

However, shapes of the probability distribution histograms varied from one corroded rebar to another, even if the average volume losses were similar, as clearly shown in Figure 8 (i.e., specimens J24 and J25 with *η*_V_ of 27.06% and 28.11%, specimens L3 and L4 with *η*_V_ of 8.07% and 8.81%). This suggests that the steel rebar corrosion is random and non-uniform, and the average corrosion degree cannot reflect the corrosion characteristics completely.

## 4. Corrosion Effects on the Tensile Behavior of Steel Rebars

### 4.1. Nominal Stress–Strain Curve

**Figure 9:**
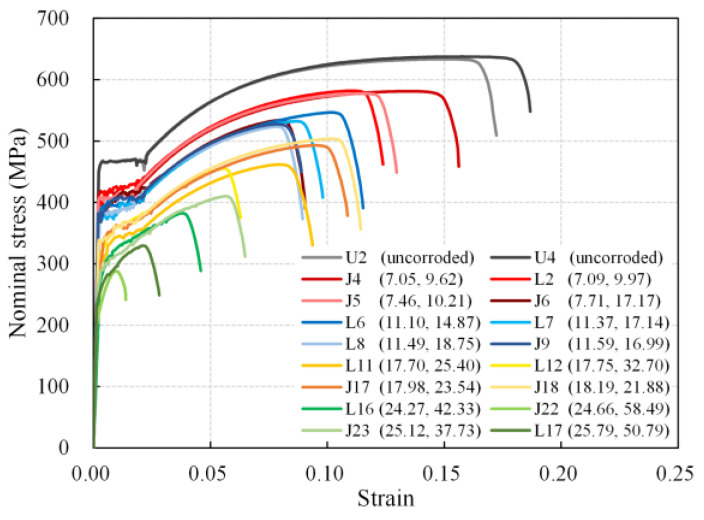
Nominal stress–strain curves of steel rebars.

The nominal stress–strain relationships derived from load-displacement curves were utilized to assess the effect of corrosion on steel rebars since the parallel length of rebar specimens may vary slightly in tensile tests. The nominal stress refers to the tensile load divided by the nominal cross-sectional area of the uncorroded rebar. The typical nominal stress–strain curves of rebars with different corrosion levels are presented in Figure 9, in which the note data are the average volume loss and maximum cross-section loss, respectively. Generally, as the average volume loss increased, both the nominal yield and the ultimate strength of the rebars decreased, and the ultimate strain (strain at the maximum load) dropped severely. The yield plateau of the curve shortened, and the boundary between the yield and the hardening stages blurred gradually with corrosion, whereas for the corroded rebar with a high average corrosion level, the yield plateau disappeared. This is consistent with the previous studies [9,12,20].

Furthermore, there are four groups of corroded rebars with similar average corrosion degrees in Figure 9, which are 7.05% to 7.71%, 11.10% to 11.59%, 17.70% to 18.19%, and 24.27% to 25.79%, respectively. By comparing the nominal stress–strain curves of four rebars in each group, it can be observed that the curves did not coincide with each other, especially at the maximum load point, and the difference tended to be more evident as the average corrosion degree grew (see Table 4). The tensile behavior diversity of rebars with similar average corrosion degrees is a result of various corrosion morphologies, suggesting that corrosion non-uniformity has an effect on the tensile behavior of corroded rebars.

### 4.2. Load-Bearing Capacity

#### 4.2.1. Tensile Test Results

**Figure 10:**
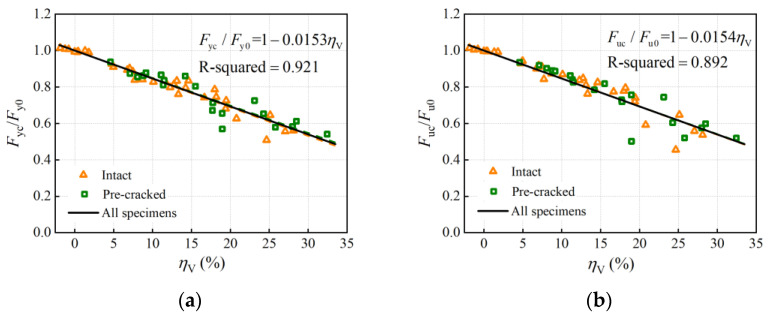
Effect of corrosion on load-bearing capacity: (**a**) yield load; (**b**) ultimate load.

The load-bearing capacity of steel rebars is typically represented by yield load and ultimate load. To evaluate the corrosion effect and compare it among rebars of different strength grades, the yield and ultimate load were normalized with respect to the corresponding uncorroded properties. Figure 10 illustrates the normalized yield and ultimate load (*F*_yc_/*F*_y0_ and *F*_uc_/*F*_u0_) as functions of average volume loss. With an increase in average volume loss, *F*_yc_/*F*_y0_ and *F*_uc_/*F*_u0_ decreased linearly, which clearly indicates that corrosion degrades the load-bearing capacity of rebar specimens. Because little difference can be seen between the data on pre-cracked and intact slabs in Figure 10, the linear regression models for the normalized yield and ultimate load can be obtained by the least square method on data from 53 tested specimens as:

(6) $$\frac{F_{yc}}{F_{y0}}=1-0.0153\eta_{V}$$

(7) $$\frac{F_{\mathrm{uc}}}{F_{u0}}=1-0.0154\eta_{V}$$

The two lines had a near-negative slope, which represents the degradation rate of the yield and the ultimate load with corrosion, indicating that the corrosion effects on the yield and ultimate bearing capacity are similar. Furthermore, it can be seen that the load-bearing capacity indices became scattered when the average corrosion level was high, possibly resulting from increasing non-uniformity of corrosion.

#### 4.2.2. Degradation Mechanism

**Figure 11:**
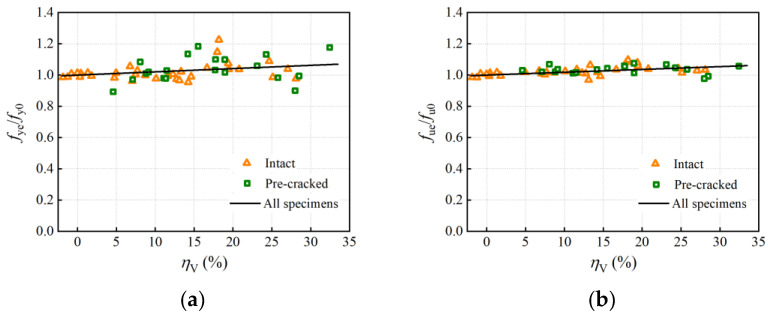
Normalized effective strength: (**a**) yield strength; (**b**) ultimate strength.

The yield and ultimate load of the corroded rebar may be influenced by the declined effective cross-sectional areas of rebar specimens and the mechanical properties of steel. In order to consider a single effect, the strength calculated on the residual cross-sectional area was proposed. Considering that corroded rebars were usually observed to damage the most severely corroded section in tensile tests and that the minimum residual cross-section (critical cross-section) distinguished from the statistically average cross-section physically exists, the effective strength of corroded rebars was computed using the minimum residual cross-sectional areas. The normalized effective yield strength (*f*_ye_/*f*_y0_) and ultimate strength (*f*_ue_/*f*_u0_) of 53 rebar specimens are plotted in Figure 11. The normalized effective yield and ultimate strength fluctuated around 1, and it seems that the effective yield and ultimate strength tended to increase slightly with the average volume loss. This implies that corrosion does not weaken the strength of the steel material.

Due to the difficulty of accurately measuring the corrosion morphology and residual cross-sections, only a few researchers in the past [12,13] studied the effective strength calculated in the minimum cross-sectional areas. Cairn et al. [13], through physical tests on plain round bars with electrical accelerated corrosion, found that yield strength calculated on the residual cross-section showed no loss while the ultimate strength revealed a small increase with the critical cross-section loss. Moreover, test results in research [12] revealed that both the yield and the ultimate strength based on the critical cross-sectional areas increased slightly with the critical cross-section loss. These findings are almost in accordance with the present study.

There may be two reasons for the increase in the effective yield and ultimate strength: On one hand, the material composition and initial defects are spatially heterogeneous throughout a steel rebar, and for an uncorroded rebar of which cross-sections are nearly uniform, it would be expected to damage the weakest position of material properties. While for a corroded rebar of which the cross-sections are apparently non-uniform, the minimum bearing cross-section may not coincide with the weakest location of the material properties [13]. When the effect of the bearing cross-section on the load-bearing capacity of the rebar specimen is greater than that of the initial uneven material properties, it will lead to a slight increase in the effective strength in contrast to the uncorroded rebar. On the other hand, severe pits caused by localized corrosion change the partial stress condition of the tensile reinforcement from an uniaxial state to a multi-axial state, which restricts the deformation development and increases the load-bearing capacity of the corroded rebars; that is, the notch-strengthening effect [43].

**Figure 12:**
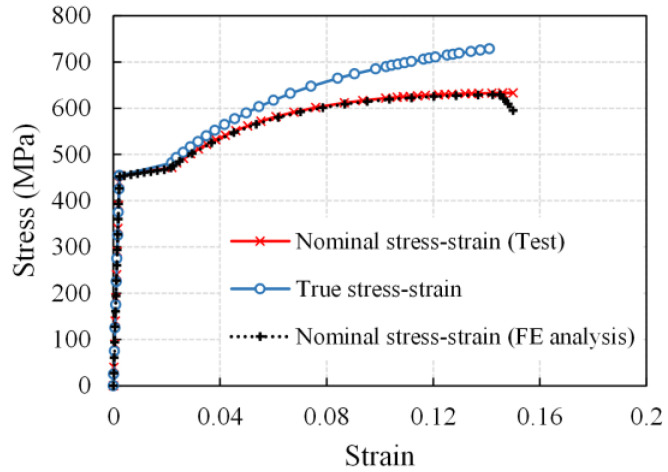
Stress–strain relation of uncorroded steel bar.

It is difficult to experimentally explore the multi-axile stress condition; hence, numerical analysis using the finite element method (FEM) was conducted by *ABAQUS* software to simulate the uniaxial tensile behavior of uncorroded bars and corroded bars with a single pit. In the FE models, a structured mesh was generated using the continuum element C3D8, of which the size ranged from 0.5 to 1.5 mm, and was refined near the corrosion pit. One end of the bar was constrained in all directions for displacement and rotation, whereas the load was applied at the other end by displacement. The true stress–strain relation, translated by the Equation (8) from the test nominal stress–strain relation of the uncorroded rebars was used to define the mechanical properties in the FE models:

(8) $$\begin{array}{l} \sigma_{\mathrm{true}}=\sigma_{n}(1+\varepsilon_{n})\\ \varepsilon_{\mathrm{true}}=\mathrm{In}(1+\varepsilon_{n}) \end{array}$$

where *σ*_true_ and *ε*_true_ are the true stress and strain that consider the actual cross-section change in tensile tests, *σ*_n_ and *ε*_n_ are the nominal stress and strain based on the original cross-sections. As shown in Figure 12, the computed nominal stress–strain curves of the uncorroded bar agreed well with the test curve, validating the FE analysis.

**Figure 13:**
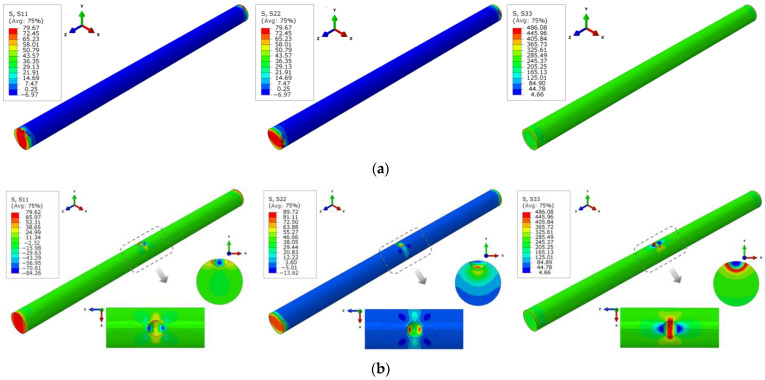
Stress nephogram in three axes of steel bars under uniaxial tensile load: (**a**) uncorroded bar; (**b**) corroded bar with a single pit.

The stress in three axes (the z-axis is along the length direction of the steel bar, the y-axis is along the direction of the erosion depth, and the x-axis is perpendicular to both the y-axis and the z-axis, in which the stress is denoted as S33, S22, and S11, respectively) distributed along the bar was obtained, as shown in Figure 13. Except for the stress concentration at the end of the bar caused by the external restraint and load, S11 and S22 of the uncorroded bar approximately equaled 0. The uncorroded bar was in the uniaxial tensile state. As for the corroded bar, S11 and S22 were significantly greater than 0 around the pit region, which is clear evidence that the corrosion pit transforms the original uniaxial stress condition into a multi-axial state. Furthermore, it should be mentioned that pit-induced stress concentration was observed merely in a limited region on the critical cross-section of the corroded bar in the FEM analysis, yet the yield load of the bar was captured when tensile stress on the cross-section completely exceeded the steel yield strength. This means that localized stress concentration resulting from corrosion pits has few influences on the load-bearing capacity of corroded rebars.

The corrosion pit leads to “notch strengthening” and stress concentration meanwhile, and there is no evidence in the literature that the material composition of corroded and uncorroded bars is different. Therefore, based on the fact that the effective strength increased a little with the cross-section loss in tensile tests, it is reasonable to assume that the effective yield and ultimate strength of corroded rebars are equal to that of the uncorroded rebar.

**Figure 14:**
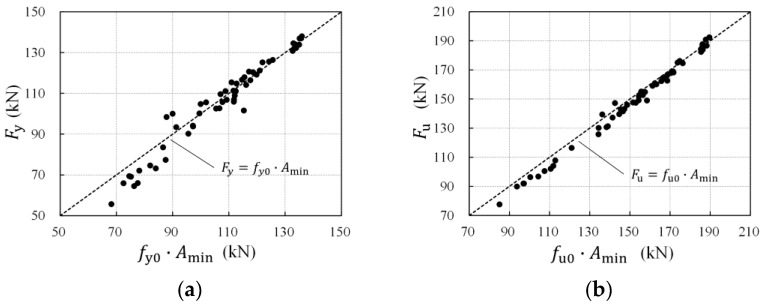
Comparison of calculation and test tensile load: (**a**) yield load; (**b**) ultimate load.

The products of the uncorroded rebar strength multiplied by the minimum cross-sectional area of the corroded rebars (*f*_y0_∙*A*_min_ and *f*_u0_∙*A*_min_) were calculated and then compared with the corresponding measured load, as shown in Figure 14. The scatter points were almost distributed near the reference line of *F*_y_ = *f*_y0_∙*A*_min_ or *F*_u_ = *f*_u0_∙*A*_min_, indicating that both the yield and the ultimate load of the corroded rebar are dominated by the minimum cross-sectional area. It can therefore be concluded that the effective critical cross-section loss is the principal cause of the corroded rebar’s degradation of the load-bearing capacity.

#### 4.2.3. Quantitative Evaluation

**Figure 15:**
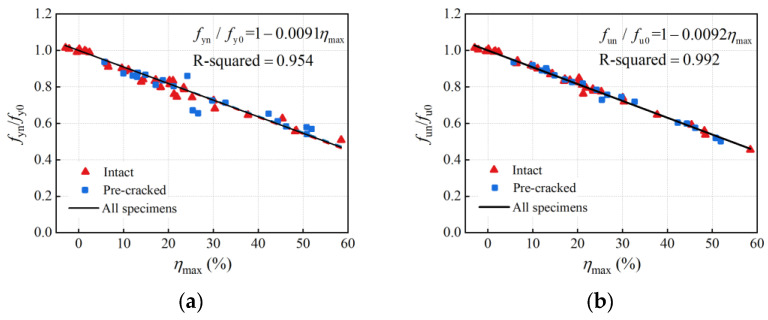
Relationships between nominal strength and maximum cross-section loss: (**a**) yield strength; (**b**) ultimate strength.

To allow application among rebars of various original diameters, the load-bearing capacity of the steel rebar is commonly represented by nominal strength rather than tensile load. The nominal strength, referring to the load per nominal cross-sectional area of the uncorroded rebar, is widely used in current research and design. Thus, the degradation relation of the load-bearing capacity was established in terms of nominal strength. As mentioned above, the residual minimum cross-sectional area corresponding to the maximum cross-section loss is the essential factor in causing load-bearing capacity deterioration. Accordingly, the relations of the nominal yield and ultimate strength to the maximum cross-section loss are presented in Figure 15, and the degradation formulae were established based on tensile test results (Equations (9) and (10)). It can be seen that both the nominal yield and the ultimate strength decreased linearly with an increase in the maximum cross-section loss, showing little data scattering with R-squared equal to 0.954 and 0.992, respectively.

(9) $$\frac{f_{yn}}{f_{y0}}=1-0.0091\eta_{\max}$$

(10) $$\frac{f_{un}}{f_{u0}}=1-0.0092\eta_{\max}$$

Compared with the relations of the yield and ultimate load to the average volume loss in Section 4.2.1, where the data points were more scattered and R-squared equaled 0.921 and 0.892 respectively, it is clear that the maximum cross-section loss is more relevant to the degradation of the load-bearing capacity. Considering that longitudinal non-uniform corrosion leads to differences in the corrosion morphology and a critical cross-section among corroded rebars with similar average corrosion levels, the maximum cross-section loss instead of the average corrosion degree (average mass, volume, or cross-section loss) adopted in degradation models of the load-bearing capacity is more accurate.

### 4.3. Ductility

#### 4.3.1. Tensile Test Results

**Figure 16:**
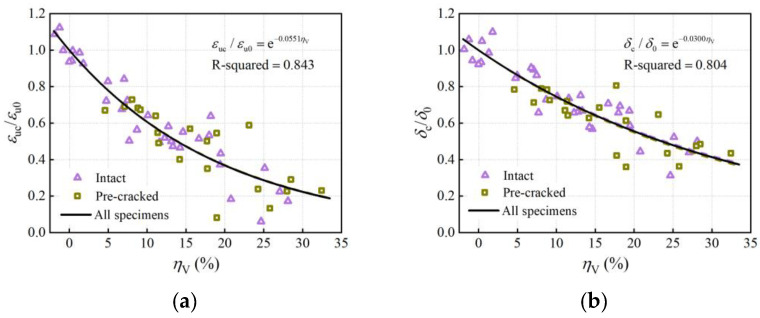
Effect of corrosion on ductility: (**a**) ultimate strain; (**b**) elongation after fracture.

A series of indices is commonly used to quantify the ductility of the steel rebar, such as ultimate strain, elongation after fracture, total energy density, and ultimate-to-yield load ratio. The ultimate strain was calculated by dividing the total elongation at the maximum load by the initial parallel length. The elongation after fracture was calculated by the ratio of the residual elongation between 11 markers near the failure area after fracture with its original gauge length. Figure 16 demonstrates the effect of corrosion on both the ultimate strain and the elongation after fracture, in which the properties were normalized by dividing it by corresponding uncorroded properties, i.e., (*ε*_uc_/*ε*_u0_) and (*δ*_c_/*δ*_0_). It can be seen that both the ultimate strain and the elongation after fracture declined significantly as the average volume loss increased. Unlike the load reduction discussed in Section 4.2.1, the two ductility indices displayed a nonlinear relationship with the average volume loss, in which they decreased rapidly at a low corrosion level, while the decline rate then slowed down slightly. Thereby, nonlinear regression analysis by the least square method was performed, and the regression models using exponential decay functions were established as:

(11) $$\frac{\varepsilon_{\mathrm{uc}}}{\varepsilon_{u0}}=\exp(-0.0551\eta_{V})$$

(12) $$\frac{\delta_{c}}{\delta_{0}}=\exp(-0.0300\eta_{V})$$

**Figure 17:**
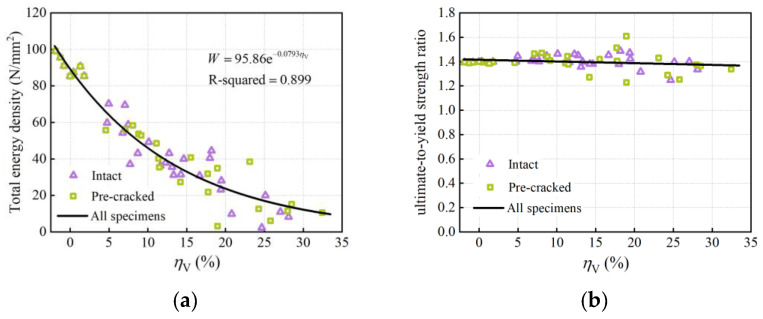
Effect of corrosion on: (**a**) energy density; (**b**) ultimate-to-yield-strength ratio.

The total energy density determined from the area under the stress–strain curve represents the energy absorption capacity of the steel rebar. Figure 17a shows the calculated total energy density as a function of the average volume loss. The total energy density also decreased nonlinearly with an increase in the average volume loss, indicating that the corrosion greatly weakens the energy absorption capacity of rebars. The exponential decay model was then established by nonlinear regression analysis. In addition, the ultimate-to-yield load ratio, a critical parameter in the seismic design of RC structures representing the post-yield security stocks, is demonstrated for 53 specimens in Figure 17b. It can be observed that the ultimate-to-yield load ratio of 53 rebars fluctuated within the range of 1.4 ± 0.2 approximately. As the average volume loss increased, it tended to decrease slightly, consistent with the findings from other literature [20,22]. Although the insignificant decrease in the ultimate-to-yield load ratio can almost be neglected, corrosion degrades rebars’ deformation capacity and energy absorption capacity after yielding, leading to brittle failure.

By comparing the corrosion effects on the load-bearing capacity and ductility in Figure 10 and Figure 16, it can be found that the ductility indices declined more rapidly with corrosion than the load-bearing capacity indices. In other words, the decay ratio of ductility was greater than that of the load-bearing capacity at a certain average corrosion level. For instance, when the average volume loss equaled 15%, the ultimate strain and elongation after fracture of the corroded rebar dropped to 44% and 64% of the uncorroded rebar, respectively, while the yield and ultimate load dropped to 77% of the uncorroded rebar, according to the empirical regression formulae in this study. This implies that the corrosion influence is more significant on the ductility of the steel rebar than on the load-bearing capacity.

#### 4.3.2. Degradation Mechanism

The correlation coefficients (R-squared) of empirical degradation relations in terms of average corrosion degrees from previous studies were collected in Table 5, together with the results in this study, to compare the relevance of various mechanical properties degradation of corroded rebars to corrosion levels. The R-squared for the ductility degradation relations is less than that for the yield and ultimate bearing capacity, irrespective of the corrosion condition, corrosion level, material type, and original diameter of the rebars. This implies that the ductility indices of the corroded rebar have less correlation with the average corrosion degree in contrast to the load-bearing capacity indices. This is because the total deformation of a rebar specimen is composed of the micro-deformation of each location within the entire parallel length of the specimen. Longitudinal non-uniform corrosion, on the other hand, contributes to a complex cross-sectional loss along the rebar, which cannot be described comprehensively by the average corrosion degree. This means that the ductility of the corroded rebar is affected not only by the average corrosion degree but also by other factors.

In order to explore the non-uniform corrosion effects on the ductility of the corroded rebar specimens, numerical analysis accumulating the elongation by each microsegment based on scanning the residual cross-sectional areas of the corroded rebar was conducted through *MATLAB*. Given that there is no literature reporting the changes in the steel material composition of a hot-rolled steel rebar with corrosion and that little difference in the effective strength of the corroded rebar and the uncorroded rebar was observed, the stress–strain relationship of the uncorroded rebar was utilized in numerical analysis to calculate the load-deformation curve of corroded rebars. The main steps in the numerical analysis were as follows:

(1) Divide the rebar specimen within its parallel length into *n* microsegments at an interval of 1 mm, assuming that the cross-sectional area of the *i*th segment is equal to *A_i_* constantly.
(2) The nonlinear strain–stress relationship of the uncorroded rebar is:

(13) $$\varepsilon =g(\sigma )=\left\{\begin{array}{l} g_{1}(\sigma ),\sigma \leq f_{y0}\\ g_{2}(\sigma ),f_{y0}<\sigma \leq f_{u0} \end{array}\right.$$

where *g*_1_(*σ*) and *g*_2_(*σ*) are regression functions of elastic and strengthening zones obtained from tensile tests. When the tensile load is *F_j_*, the stress and strain of the *i*th segment are:

(14) $$\sigma_{ij}=F_{j}/A_{i}$$

(15) $$\varepsilon_{ij}=g(\sigma_{ij})=g(F_{j}/A_{i})$$

(3) The total deformation of a rebar specimen ∆*l_j_* under the tensile load of *F_j_* can then be accumulated by each microsegment as:

(16) $$\Delta l_{j}=\sum_{i=1}^{n}\Delta l_{ij}=\sum_{i=1}^{n}\varepsilon_{ij}\cdot 1=\sum_{i=1}^{n}g(F_{j}/A_{i})$$

(4) Compute the deformation under successive increments of the tensile load; finally, the load-deformation curve can be obtained.

It should be noted that this numerical analysis can only simulate the load-deformation curve before necking for the reason that the deformation grows evenly along the rebar before the necking stage in tensile tests, while severe plastic deformation proliferates around the critical cross-section and the rebar thins locally when the tensile load is beyond the critical value. Accordingly, the maximum load in the calculation should not be larger than the ultimate load which is measured in tensile tests or estimated by degradation models.

**Figure 18:**
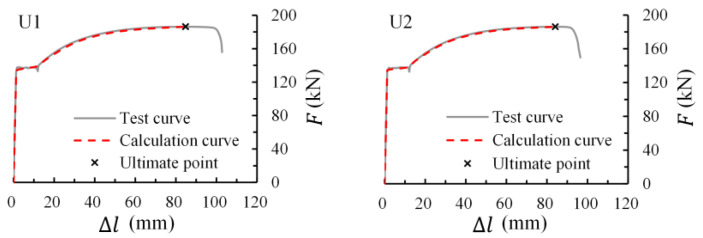
Calculation load-deformation curves of uncorroded rebars.

Figure 18 shows the application of numerical analysis in uncorroded rebars of this study. The deformation ratio between calculation and test values at the maximum load were 0.980 and 1.017 for specimens U1 and U2. Almost no deviation between calculation and test load-deformation curves could be observed, which validates the numerical method in simulating the tensile behavior of rebars before necking.

**Figure 19:**
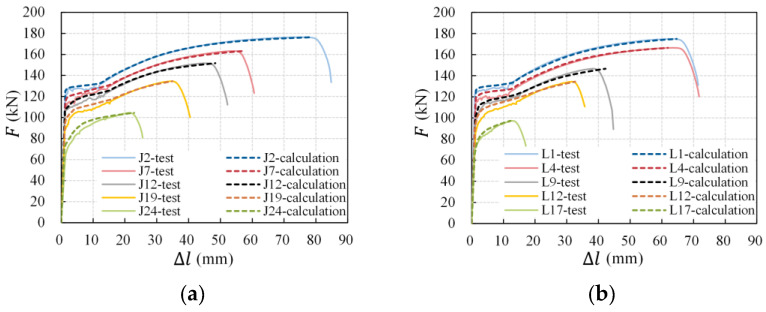
Calculation of load-deformation curves of corroded rebars: (**a**) intact slabs; (**b**) pre-cracked slabs.

The tensile behavior of longitudinal non-uniform corroded rebars was then simulated. A representative comparison between the calculation and test results of specimens with different average corrosion levels is demonstrated in Figure 19 and Table 6. It can be seen that both the shape and the critical points (yield and ultimate point) of the calculation load-deformation curves were in good agreement with the corresponding test curves, and the deviations in tensile deformation at the maximum load calculated on scanning areas were within 10%, indicating that the numerical method accurately simulates the effect of longitudinal non-uniform corrosion on the tensile behavior of corroded rebars. Furthermore, considering that the numerical method is based on the mechanical properties of an uncorroded rebar and residual cross-sectional areas of corroded rebars, this result implies that steel properties are unaffected by corrosion and that the cross-section loss is the principal cause of the ductility degradation of corroded rebars. The decrease in ductility indices is associated with the cross-section loss along the entire length of the corroded rebars.

#### 4.3.3. Quantitative Estimation

**Figure 20:**
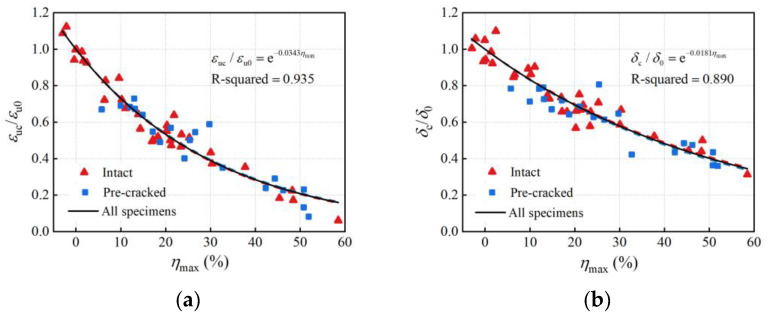
Relationships between ductility indices and maximum cross-section loss: (**a**) ultimate strain; (**b**) elongation after fracture.

The critical cross-section primarily determines the ultimate load and fracture timing in tensile tests and, thus, is closely associated with the rebar deformation. Figure 20 illustrates the relationships between the maximum cross-section loss and the normalized ultimate strain and elongation after fracture. Through regression analysis, the ductility degradation models in terms of nonlinear function were proposed as:

(17) $$\frac{\varepsilon_{uc}}{\varepsilon_{u0}}=\exp(-0.0343\eta_{\max})$$

(18) $$\frac{\delta_{c}}{\delta_{0}}=\exp(-0.0181\eta_{\max})$$

In comparison to the relations in Figure 16 and Equations (11) and (12), the decreased ductility indices elicited relatively stronger correlations with the maximum cross-section loss than with the average volume loss (R-squared based on the maximum cross-section loss were 0.935 and 0.890, and R-squared based on the average volume loss were 0.843 and 0.804). The ultimate strain represents the deformation capacity under the ultimate load. The elongation after fracture contains the maximum uniform plastic deformation. Therefore, using the maximum cross-section loss to assess the corrosion effect on the ultimate strain and elongation after fracture achieves higher accuracy than using the average corrosion degree.

Moreover, the “microsegment accumulation” numerical method in Section 4.3.2, taking into account the cross-section loss of the entire length of the rebar, is an efficient approach to simulate the non-uniform corrosion effect on the tensile behavior of corroded rebars. The difficulty of measuring the corrosion patterns and residual cross-sectional areas of the whole rebar in engineering may prevent the application of the numerical method. Therefore, based on the statistical characteristics of cross-sections in Section 3.3, a normal distribution model of residual cross-sectional areas with equivalent mean and variance to scanning corrosion morphology data was established for the numerical analysis. The probability density function can be expressed as:

(19) $$f(A_{i})=\frac{1}{S\sqrt{2\pi }}\exp\left[-\frac{{\left(A_{i}-\mu \right)}^{2}}{2S^{2}}\right]$$

where *f*(*A_i_*) is the probability density of the cross-sectional area *A_i_*, *μ* is the mean value, and *S*^2^ is the variance which can be evaluated by a regression formula in Section 3.2. Although the Weibull distribution model has a little advantage in describing the non-symmetrical distribution of cross-sectional areas [44], its three parameters make the model complicated and difficult to apply. The normal distribution with a simple and practical pattern is widely used, and its two parameters (*μ* and *S*^2^) respectively represent the average corrosion level and longitudinal corrosion non-uniformity. Accordingly, the normal distribution is reasonable to characterize the cross-sectional areas of rebar specimens in the numerical analysis for rebar tensile behavior. The equivalent probability density distributions of cross-sectional areas for representative rebars with various corrosion levels are shown as blue curves in Figure 8.

**Figure 21:**
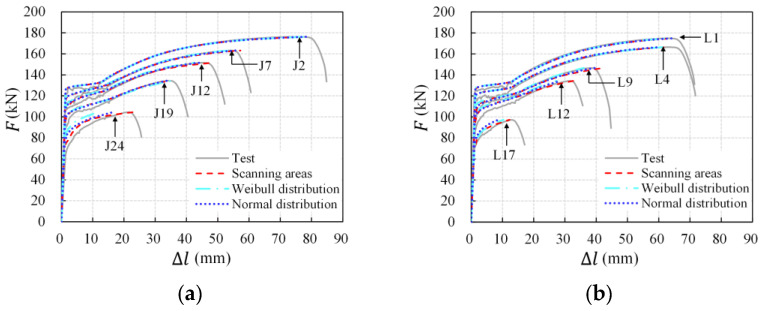
Calculation of load-deformation curves of corroded rebars based on simulating cross-sectional areas: (**a**) intact slabs; (**b**) pre-cracked slabs.

Rebar cross-sectional area data were then acquired by the random number generation command of *MATLAB* according to the equivalent normal distribution model and were utilized in numerical calculations for tensile behavior. Weibull distribution models were also adopted to be compared. Calculation load-deformation curves and deformation values based on scanning and simulating cross-sectional areas were compared with experimental curves in Figure 21 and Table 6. The majority of the simulating results using the simplified distribution (Weibull and normal distributions) of cross-sectional areas were in good agreement with the test results and the calculation results based on the actual corrosion morphology, except for rebars with an average volume loss larger than 20%. The deviation for the high corroded rebar in the ultimate strain was contributed by the difference in cross-sectional area distributions between the simplified symmetric unimodal model and the actual multimodal or significant left-skewed patterns. Furthermore, the performance of the Weibull distribution and normal distribution in estimating tensile properties of corroded rebars showed little difference in general. In conclusion, the equivalent distribution model of the rebar cross-sectional areas and the “microsegment accumulation” numerical method in the present study are capable of predicting the tensile behavior of the corroded rebar with an average corrosion degree of less than 20%.

## 5. Conclusions

In this study, non-uniform corroded rebars were acquired by an electrical corrosion experiment coupled with a specific cathode arrangement for several partial-immersed pre-cracked and intact RC slabs. On the basis of residual cross-section data measured by 3D laser scanning, the characteristics of longitudinal non-uniform corrosion were investigated. The tensile behavior of non-uniform corroded rebars was studied by uniaxial tensile tests and numerical analysis. The following conclusions can be drawn:

1. The corrosion location, range, and localized corrosion levels of embedded rebars were stochastic and longitudinally non-uniform for both pre-cracked and intact RC slabs since they were affected not only by cracks but also by the uneven RC composition and variable environmental conditions. Characterized by the maximum cross-section loss and the variance in the cross-sectional areas, the corrosion non-uniformity along the length of the rebar increases with the increasing average corrosion degrees.
2. The probability distribution of cross-sectional areas for uncorroded and slightly corroded rebars was generally unimodal. As the average corrosion degree increased, it changed from symmetrical to left-skewed. When the average volume loss was greater than 20%, a multimodal distribution for the cross-sectional areas could be observed.
3. The corrosion level affects the nominal stress–strain curves of the corroded steel rebars in terms of the yield limit, ultimate limit, and yield plateau. On the other hand, corrosion non-uniformity also has non-negligible effects on it; differences were found between corroded rebars with similar average corrosion levels, especially around the point of ultimate limit.
4. The yield and ultimate load decreased linearly with an increase in the average volume loss. However, corrosion did not weaken the effective yield and ultimate strength calculated on the minimum residual cross-sectional areas. The strengthening effect resulting from corrosion pits even slightly increased the effective strength of the corroded rebars. The main cause of the load-bearing capacity degradation of corroded rebars is the loss of the effective critical cross-section, and the nominal strength of the steel rebars is dominated by the critical cross-sectional areas. Thus, by considering the longitudinal non-uniform corrosion, it is more accurate to adopt the maximum cross-section loss instead of the average corrosion degree to estimate the corroded rebars’ degraded load-bearing capacity.
5. The ultimate strain and elongation after fracture decreased exponentially with an increase in average volume loss. The degradation models based on the maximum cross-section loss were found to be better correlated with the test results than those based on the average volume loss. The ductility of the corroded rebars decreased more rapidly and was affected by more factors compared with the load-bearing capacity. Cross-sectional loss along the entire length of the rebar is the primary cause of ductility degradation. The mechanical properties of steel are unaffected.
6. From the numerical calculation of corroded rebars based on the mechanical properties of the uncorroded rebar and scanning the cross-sectional areas of the corroded rebar, the calculation of load-deformation curves agreed with the test curves. Normal distribution models, in which the mean and variance are equivalent to practical values, were proposed to simulate the cross-sectional areas of corroded rebars for numerical analysis.

## Figures and Tables

**Table 1:** Summary of past experimental studies on tensile properties of corroded rebars.

Author	Diameter (mm)	Specimens Condition	Corrosion Process	Degradation of Load-Bearing Capacity	Degradation of Ductility
Vanama et al. [9]	Φ12.7 (MS 250, MS 350)	Bars in concrete	Natural (Service)	$f_{\mathrm{yc}}=f_{y0}(1-0.0122\eta )$ $f_{\mathrm{uc}}=f_{u0}(1-0.0119\eta )$	$\varepsilon_{\mathrm{uc}}=\varepsilon_{u0}\cdot e^{-0.0292\eta }$
Ou et al. [18]	D13, D16, D19	Bars in concrete	Natural (Service)	$f_{\mathrm{yc}}=f_{y0}(1-0.0123\eta )$ $f_{\mathrm{uc}}=f_{u0}(1-0.0115\eta )$	$\varepsilon_{\mathrm{uc}}=\varepsilon_{u0}(1-0.0125\eta )$
Lee and Cho [16]	D10, D13 (SD295A)	Bars in concrete	Artificial (Wet and Dry)	$f_{\mathrm{yc}}=f_{y0}(1-0.0198\eta )$ $f_{\mathrm{uc}}=f_{u0}(1-0.0157\eta )$	$\delta_{c}=\delta_{0}(1-0.0259\eta )$
Lu et al. [17]	D16 (HRB400)	Bars in concrete	Artificial (Wet and Dry)	$f_{\mathrm{yc}}=f_{y0}(1-0.0195\eta )$ $f_{\mathrm{uc}}=f_{u0}(1-0.0231\eta )$	$\delta_{c}=\delta_{0}(1-0.0460\eta )$
Cairns et al. [13]	Φ16	Bars in concrete	Electrical (Wet and Dry)	$f_{\mathrm{yc}}=f_{y0}(1-0.0120\eta )$ $f_{\mathrm{uc}}=f_{u0}(1-0.0110\eta )$	$\varepsilon_{\mathrm{uc}}=\varepsilon_{u0}(1-0.0300\eta )$
Tang et al. [12]	D19.1 (Grand420)	Bars in concrete	Electrical (Salt spray)	$F_{\mathrm{yc}}=F_{y0}(1-0.0170\eta )$ $F_{\mathrm{uc}}=F_{u0}(1-0.0170\eta )$	$\Delta l_{u}=6.37+12.30\cdot e^{-\eta /10.42}$
Ou et al. [18]	D13, D29 (A706)	Bars in concrete	Electrical (Full-immersed)	$f_{\mathrm{yc}}=f_{y0}(1-0.0127\eta )$ $f_{\mathrm{uc}}=f_{u0}(1-0.0116\eta )$	$\varepsilon_{\mathrm{uc}}=\varepsilon_{u0}(1-0.0281\eta )$
Lee and Cho [16]	D13 (SD295A, SD345D)	Bars in concrete	Electrical (Full-immersed)	$f_{\mathrm{yc}}=f_{y0}(1-0.0124\eta )$ $f_{\mathrm{uc}}=f_{u0}(1-0.0107\eta )$	$\delta_{c}=\delta_{0}(1-0.0195\eta )$
Kashani et al. [15]	D8, D12 (B500B, B500)	Bars in concrete	Electrical (Full-immersed)	$f_{\mathrm{yc}}=f_{y0}(1-0.0170\eta )$ $f_{\mathrm{uc}}=f_{u0}(1-0.0180\eta )$	N/S
Zhang et al. [10]	N/S (HPB235, HRB335, HRB400)	Bars in concrete	Electrical (Half-immersed)	$F_{\mathrm{yc}}=F_{y0}(1-\beta_{y}\eta )$ $\beta_{y}=0.0177,\;0.0105,\;0.0106$ $F_{\mathrm{uc}}=F_{u0}(1-\beta_{u}\eta )$ $\beta_{u}=0.0245,\;0.0106,\;0.0119$	$\varepsilon_{\mathrm{uc}}=\varepsilon_{u0}\cdot e^{-0.0813\eta }$ $\varepsilon_{\mathrm{uc}}=\varepsilon_{u0}\cdot e^{-0.0550\eta }$
Xia et al. [20]	D16, D20 (HRB335, HRB500)	Bars in concrete	Electrical	$f_{\mathrm{yc}}=f_{y0}(1-0.0210\eta )$ $f_{\mathrm{uc}}=f_{u0}(1-0.0210\eta )$	$\delta_{c}=\delta_{0}(1-0.0800\eta )$
Sun et al. [19]	D14, D16 (HRB400, HRB500)	Bare bars on wet sponge	Electrical	$f_{\mathrm{yc}}=f_{y0}(1-0.0110\eta )$ $f_{\mathrm{uc}}=f_{u0}(1-0.0130\eta )$	$\delta_{c}=\delta_{0}(1-0.0413\eta_{\max})$
Xia et al. [20]	Φ6, Φ8, Φ10 D12, D14, D16, D20	Bare bars on wet sponge	Electrical	$f_{\mathrm{yc}}=f_{y0}(1-0.0120\eta )$ $f_{\mathrm{uc}}=f_{u0}(1-0.0120\eta )$	$\delta_{c}=\delta_{0}(1-0.0200\eta )$
Vanama et al. [9]	D16 (Fe 500D)	Bare bars	Electrical (Half-immersed)	$f_{\mathrm{yc}}=f_{y0}(1-0.0136\eta )$ $f_{\mathrm{uc}}=f_{u0}(1-0.0128\eta )$	N/S
Imperatore et al. [14]	D8, D12, D16, D20 (S500C)	Bare bars	Electrical (Half-immersed)	$f_{\mathrm{yc}}=f_{y0}(1-0.0143\eta )$ $f_{\mathrm{uc}}=f_{u0}(1-0.0186\eta )$	$\varepsilon_{\mathrm{uc}}=\varepsilon_{u0}\cdot e^{-0.0205\eta }$

Notes: D-Ribbed steel rebar; Φ-Plain steel bar; and N/S = not stated.

**Table 2:** Steel characterization of HRB400 rebars.

*d*_n_ (mm)	*f*_y0_ (MPa)	*f*_u0_ (MPa)	*E*_0_ (MPa)	*ε*_u0_	*δ*_0_
20	465.00	648.17	2.02 × 10^5^	0.159	0.254

**Table 3:** Average volume loss, average cross-section loss, maximum cross-section loss.

No.	*η*_V_ (%)	*η*_avg_ (%)	*η*_max_ (%)	No.	*η*_V_ (%)	*η*_avg_ (%)	*η*_max_ (%)
U1	−0.01	0.10	1.65	J1	4.75	4.86	6.35
U2	0.32	0.44	−0.38	J2	4.96	5.07	6.59
U3	0.43	0.54	−0.07	J3	6.76	6.86	11.08
U4	1.32	1.43	1.35	J4	7.05	7.15	9.62
U5	1.80	1.91	2.40	J5	7.46	7.41	10.21
U6	−1.86	−2.11	−2.94	J6	7.71	7.81	17.17
U7	−1.25	−1.24	−2.13	J7	8.70	9.11	14.35
U8	−0.79	−1.07	0.12	J8	10.11	10.04	13.92
L1	4.59	4.70	5.73	J9	11.59	11.68	16.99
L2	7.09	7.19	9.97	J10	12.26	12.35	18.28
L3	8.07	8.17	12.99	J11	12.75	12.84	20.30
L4	8.81	8.91	12.03	J12	13.11	13.21	21.02
L5	9.15	9.26	13.15	J13	13.32	13.40	21.25
L6	11.10	11.04	14.87	J14	14.24	14.34	23.41
L7	11.37	11.47	17.14	J15	14.62	14.72	20.22
L8	11.49	11.59	18.75	J16	16.66	16.75	25.28
L9	14.20	14.30	24.22	J17	17.98	18.07	23.54
L10	15.51	15.61	21.15	J18	18.19	18.28	21.88
L11	17.70	17.79	25.40	J19	19.39	19.47	30.35
L12	17.75	17.83	32.70	J20	19.46	19.54	30.06
L13	18.96	18.90	51.91	J21	20.79	20.87	45.40
L14	18.95	19.03	26.60	J22	24.66	24.73	58.49
L15	23.11	23.20	29.78	J23	25.12	25.18	37.73
L16	24.27	24.35	42.33	J24	27.06	27.14	48.25
L17	25.79	26.80	50.79	J25	28.11	28.17	48.47
L18	28.00	28.07	46.22				
L19	28.50	28.57	44.35				
L20	32.45	32.51	50.83				

Notes: *η*_V_, *η*_avg_, and *η*_max_ were calculated on the basis of the corresponding mean value of eight uncorroded specimens. Thus, the calculation values of some uncorroded rebars were negative.

**Table 4:** Comparison of tensile properties of corroded rebars with similar average corrosion degrees.

No.	*η*_V_ (%)	*f*_yn_ (MPa)	*f*_un_ (MPa)	*ε*_uc_	No.	*η*_V_ (%)	*f*_yn_ (MPa)	*f*_un_ (MPa)	*ε*_uc_
J4	7.05	410.35	581.84	0.134	L11	17.70	305.32	462.26	0.080
L2	7.09	397.49	582.45	0.110	L12	17.75	324.21	455.78	0.056
J5	7.46	402.38	578.01	0.115	J17	17.98	357.67	493.50	0.085
J6	7.71	381.45	534.52	0.080	J18	18.19	339.08	504.29	0.102
L6	11.10	394.00	547.27	0.102	L16	24.27	296.93	382.91	0.038
L7	11.37	369.00	532.75	0.087	J22	24.66	231.37	288.63	0.010
L8	11.49	380.16	523.93	0.078	J23	25.12	293.74	410.45	0.056
J9	11.59	378.60	528.00	0.079	L17	25.79	263.26	329.95	0.021

**Table 5:** Summary of correlation between mechanical characteristics and average corrosion degree.

Author	Corrosion Condition	R-Squared		
		Yield Bearing Capacity	Ultimate Bearing Capacity	Ductility
Vanama et al. [9]	Natural (Service)	0.981	0.969	0.883
Ou et al. [18]	Natural (Service)	0.938	0.948	0.628
Lee and Cho [16]	Artificial (Wet and Dry)	0.924	0.891	0.842
Ou et al. [18]	Electrical (Full-immersed)	0.777	0.811	0.392
Lee and Cho [16]	Electrical (Full-immersed)	0.946	0.973	0.782
Tang et al. [12]	Electrical (Salt spray)	0.850	0.880	0.400
Imperatore et al. [14]	Electrical (Half-immersed)	0.917	0.936	0.903
This study	Electrical (Half-immersed)	0.921	0.892	0.843, 0.804

Notes: Yield- and ultimate bearing capacity were denoted by load or nominal strength, and the ductility was denoted by the ultimate strain, deformation at maximum load, or elongation after fracture.

**Table 6:** Tensile deformation at the maximum load based on various cross-sectional area data.

No.	*η*_V_ (%)	∆*l*_u_ Test Value (mm)	∆*l*_u_ Calculation Value (mm)			Calculation Deviation (%)		
			Scanning Areas	Weibull	Normal	Scanning Areas	Weibull	Normal
J2	4.96	76.33	78.58	78.42	78.62	2.94	2.73	2.99
J7	8.70	54.10	57.26	53.57	55.76	5.84	−0.98	3.05
J12	13.11	45.50	48.38	42.50	44.23	6.32	−6.61	−2.79
J19	19.39	34.45	34.93	35.39	33.92	1.40	2.73	−1.54
J24	27.06	21.57	22.89	11.69	16.36	6.11	−45.82	−24.15
L1	4.59	63.22	64.67	63.11	64.33	2.29	−0.18	1.75
L4	8.81	62.85	61.89	61.26	60.21	−1.54	−2.52	−4.20
L9	14.20	38.50	42.19	35.99	39.58	9.58	−6.54	2.80
L12	17.75	31.66	32.67	27.35	28.66	3.20	−13.62	−9.47
L17	25.79	12.85	12.25	10.30	8.64	−4.63	−19.79	−32.72

## Data Availability

Some data that support the findings of this study are available from the corresponding author upon reasonable request.

## Notations

$F_{y0}$	Yield load of uncorroded rebar	$\eta_{V}$	Average volume loss
$F_{u0}$	Ultimate load of uncorroded rebar	$\eta_{\mathrm{avg}}$	Average cross-section loss
$F_{\mathrm{yc}}$	Yield load of corroded rebar	$\eta_{\max}$	Maximum cross-section loss
$F_{u0}$	Ultimate load of corroded rebar	$A_{0,avg}$	Average cross-sectional areas of uncorroded rebar
$f_{y0}$	Yield strength of uncorroded rebar	$A_{0,\min}$	Minimum cross-sectional areas of uncorroded rebar
$f_{u0}$	Ultimate strength of uncorroded rebar	$A_{c,avg}$	Average cross-sectional areas of corroded rebar
$f_{\mathrm{yc}}$	Yield strength of corroded rebar	$A_{c,\min}$	Minimum cross-sectional areas of corroded rebar
$f_{\mathrm{uc}}$	Ultimate strength of corroded rebar	$V_{0}$	Volume of uncorroded rebar
$\varepsilon_{u0}$	Ultimate strain of uncorroded rebar	$V_{c}$	Volume of corroded rebar
$\varepsilon_{\mathrm{uc}}$	Ultimate strain of corroded rebar	l	Parallel length of tensile rebar
$\delta_{0}$	Elongation after fracture of uncorroded rebar	$f_{\mathrm{yn}}$	Nominal yield strength (*F*_y_/*A*_n_)
$\delta_{c}$	Elongation after fracture of corroded rebar	$f_{\mathrm{un}}$	Nominal ultimate strength (*F*_u_/*A*_n_)
$\Delta l_{u}$	Deformation at maximum load	$f_{\mathrm{ye}}$	Effective yield strength (*F*_y_/*A*_min_)
η	Average corrosion degree (mass or volume loss)	$f_{\mathrm{ue}}$	Effective ultimate strength (*F*_u_/*A*_min_)
$d_{n}$	Nominal (original) diameter	σ	Stress
$A_{n}$	Nominal (original) cross-sectional areas	ε	Strain
$E_{0}$	Elastic modulus of uncorroded rebar		
